# Efficacy, Safety, and Cost-Effectiveness of “Internet + Pharmacy Care” Via the Alfalfa App in Warfarin Therapy Management After Cardiac Valve Replacement: Randomized Controlled Trial

**DOI:** 10.2196/53586

**Published:** 2025-05-20

**Authors:** Yiyi Qian, Weizhao Chen, Bin Zhou, Jiangya Li, Yuanyuan Guo, Zhiying Weng, Jinhua Zhang

**Affiliations:** 1 Clinical Research and Trial Center, The Second Affiliated Hospital of Kunming Medical University, 1st floor, Building 1, Inpatient Building, #374 Dianmian Road, Kunming, 650000, China, 86 13708861691; 2 The Second Clinical College of Kunming Medical University, Kunming, China; 3 School of Pharmacy, Kunming Medical University, Yunnan, Kunming, China; 4 Department of Vascular Surgery, Fuwai Yunnan Hospital, Chinese Academy of Medical Sciences, Yunnan, Kunming, China; 5 Department of Pharmacy, Fujian Maternity and Child Health Hospital College of Clinical Medicine for Obstetrics and Gynecology and Pediatrics, Fuzhou, China

**Keywords:** smartphone, mobile phone, alfalfa app, warfarin, anticoagulation, internet + pharmacy care

## Abstract

**Background:**

Anticoagulation management is important in preventing complications in patients undergoing cardiac valve replacement. The development of mobile apps offers new opportunities for the management of long-term anticoagulants. However, there is a lack of randomized controlled trials evaluating the effectiveness, safety, cost-effectiveness, and user demand for internet-based anticoagulation management.

**Objective:**

This study aimed to evaluate the efficacy, safety, and cost-effectiveness of a 3-month warfarin dose adjustment mobile app Alfalfa compared to offline management in patients postcardiac valve replacement. We also explored the app’s feasibility on user satisfaction and demand.

**Methods:**

This study was a randomized controlled trial with assessments conducted at baseline and at a 3-month follow-up. Participants were eligible if they had been on warfarin therapy for at least 3 months, received warfarin management either through the Alfalfa app or through pharmacist-led anticoagulation outpatient clinic visits, consented to regular follow-ups, and had not experienced serious bleeding or thrombotic events in the 3 months before warfarin treatment. A *P* value of ≤.05 was considered statistically significant.

**Results:**

A total of 405 participants were included in the analysis. The time in therapeutic range was significantly higher in the Alfalfa app group than in the offline group (66.46% vs 46.65%, *P*<.001). Participants in the Alfalfa app group had a higher monitoring frequency (8.14 vs 4.47, *P*<.001) and a greater percentage of international normalized ratio values within the target range (896/1660, 53.98% vs 346/899, 38.49%; *P*<.001) than those in the offline group. In addition, the Alfalfa app group exhibited lower rates of subtherapeutic (235/1660, 14.16% vs 152/899, 16.91%; *P*<.05) and extreme subtherapeutic international normalized ratio values (273/1660, 16.45% vs 186/899, 20.69%; *P*<.05) than the offline group. However, the incidence of minor bleeding was higher in the Alfalfa app group (12/204, 5.9% vs 3/201, 1.5%; *P*=.02). In terms of cost-effectiveness, the Alfalfa app group had a significantly lower average cost per test (42.37 vs 78.3, *P*<.001), average time per test (47.42 vs 90.74, *P*<.001), and cost-effectiveness ratio (385.9 vs 662.9) than the offline group. A total of 86 participants completed the satisfaction questionnaire, and the vast majority of participants expressed high levels of satisfaction with the Alfalfa App, while also providing further suggestions for improvement.

**Conclusions:**

The integration of “Internet+Pharmacy Care” using the Alfalfa App can improve the effectiveness of warfarin anticoagulation management in patients following heart valve surgery. The Alfalfa app provides a more efficient, secure, and cost-effective solution to warfarin management than traditional offline methods.

## Introduction

Valvular heart disease is one of the most prevalent cardiac conditions in China. According to 2019 extracorporeal circulation statistics, the number of heart valve surgeries continues to rise, with approximately 73,000 procedures performed annually, accounting for nearly 30% of all cardiac surgeries [1,2]. The primary treatment modalities for valvular heart disease include artificial valve replacement and valve repair surgery, both of which require long-term oral anticoagulant therapy postoperatively. Warfarin, a traditional oral anticoagulant, which widely used in clinical practice, remains the primary medication for reducing the risk of thrombosis and stroke after heart valve replacement surgery [3-5]. Patients undergoing biological valve replacement typically require 3 to 6 months of oral warfarin therapy, while those with mechanical valves need lifelong anticoagulation. Managing warfarin therapy poses challenges due to its narrow therapeutic range, interactions with food and drugs, and significant interindividual variability in dosage requirements. These challenges have been observed both domestically and internationally [6-8].

In recent years, anticoagulation clinics and home-based follow-up management models have been established in various countries. In developed regions of China, pharmaceutical services led by pharmacists in hospitals have moderately enhanced the effectiveness of anticoagulation treatment. However, both globally and in China, issues persist regarding the low efficiency and high use of human and material resources in postoperative anticoagulation management for patients with valve disease [6,9]. Many patients receiving oral anticoagulation therapy reside in rural areas where access to quality medical resources is limited. Even urban patients often face constraints such as doctor-to-patient ratios and limited consultation time. Outpatient physicians always struggle to provide comprehensive medication guidance and follow-up for every oral anticoagulation therapy patient, which can negatively impact the long-term treatment outcomes for these individuals [10,11].

With the development of information technology, countries worldwide are actively exploring personalized, intelligent and efficient eHealth tools based on their own anticoagulation management models. Some guidelines emphasize the potential of eHealth tools in improving anticoagulation management and medical outcomes [12]. The use of these eHealth tools to assist physicians with anticoagulation decision-making is recommended [13]. In April 2018, the General Office of the State Council of China issued the “Opinions on Promoting the Development of “Internet+Healthcare,” which clearly supports medical institutions in providing telemedicine services and promoting effective communication between doctors and patients.

In 2020, our research team developed an app called Alfalfa for warfarin dose adjustment [14], which has demonstrated significant advantages compared with other eHealth tools in China [15]. Through the app, patients can report coagulation results and health status to their doctors or pharmacists through mobile devices. Medication is then adjusted based on the recommendations, and international normalized ratio (INR) values are monitored accordingly [14]. Our previous retrospective observational cohort study explored the safety and efficacy of warfarin anticoagulation management through the Alfalfa App. The study confirmed that patients who use the app to manage experienced improved anticoagulation quality, reduced major bleeding events, and lower emergency and hospitalization rates compared with those receiving traditional offline management. This model has been demonstrated to be particularly suitable for patients in rural and remote areas [15].

Based on the previous study and the “Internet+pharmaceutical service” model we established, we further designed this prospective randomized controlled study in the Yunnan region of western China. The aim is to further investigate the safety, effectiveness, and cost-efficiency of the Alfalfa app in managing warfarin anticoagulation in patients with valvular disease and to better understand the app’s usage requirements.

## Methods

### Study Design

This study was a single-center randomized control trial (trial registration: ChiCTR1900021920) with assessments conducted at baseline and post intervention (3 months). Participants randomized to the intervention group received anticoagulation management through the Alfalfa App (“Internet+pharmacy care”), while the control group received offline management at a pharmacist-led anticoagulation clinic. Both groups were followed up for 3 months.

### Sample Size

The primary outcome indicator, time in therapeutic range (TTR), was used to calculate the required sample size. Our previous research suggested that TTR for the web-based and offline groups was 73% and 60%, respectively [7]. With a power of 80% and a significance level of .05, 1:1 ration of test and control groups, a minimum of 201 patients per group is required, calculated using PASS 27 software. Assuming 10% dropout rate, each group needed at least 223 patients, for a total of 446 patients.

### Recruitment and Selection

Participants were recruited from Fuwai Yunnan Cardiovascular Hospital. Patients diagnosed with valvular heart disease, who underwent valve surgery at the hospital received warfarin education from clinical pharmacists before discharge. After training, a validated questionnaire [16] was used to assess the patient’s understanding of warfarin management. If patients missed a question, they would be retrained and answered until all the questions were answered correctly. These patients were eligible for the study if they had been on warfarin treatment for at least 3 months, received warfarin management through either the Alfalfa app or pharmacist-led outpatient clinic visits, agreed to regular follow-up, had no serious bleeding or thrombotic events in the 3 months before warfarin treatment. The exclusion criteria included pregnancy, switching to another anticoagulant during the study period, and having 3 or fewer INR records during the follow-up period. After obtaining informed consent and completing the baseline questionnaire, participants were randomized into either the web-based group or offline group.

### Randomization and Blinding

Participants were randomized using a Microsoft Excel–generated randomization list in a 1:1 ratio. After participants completed baseline assessments, a research team member (unblinded) assigned participants to a group based on the randomization list. Due to the nature of the intervention, the study was open labeled and blinding was not feasible.

### Web-Based Group (Intervention Group）

After randomization, participants in the web-based group were instructed through WeChat (Tencent) to download and install the Alfalfa app on their smartphones. Detailed instructions on app usage were provided. The Alfalfa app (Alfalfa Health Management) was used for remote warfarin management in the web-based group; a detailed description of the app has been published earlier [14,15]. Patients selected a pharmacist within the app who would manage their warfarin dosage. The chosen pharmacist reviewed INR results uploaded by the patient and provided recommendations on dosage adjustments and the timing of subsequent INR tests. The coagulation management team consist of 4 cardiologists and clinical pharmacists specializing in anticoagulation from Yunnan Cardiovascular Hospital. A senior clinical pharmacist responded to patient queries within 30 minutes of receiving uploads.

### Offline Group (Control Group)

Participants in the offline group were instructed to visit the pharmacist’s office for periodic INR testing. Warfarin dosage adjustments were supervised by the same senior clinical pharmacist who managed the web-based group.

### INR Testing and Warfarin Dose Adjustment

All participants adjusted their warfarin dosage as needed and underwent periodic INR testing throughout the study. Participants in the web-based group could test INR at nearby hospitals and receive adjustment suggestions from clinical pharmacists through the app. Patients in the offline group were required to visit the hospital’s anticoagulation clinic. Warfarin dose adjustment protocols were standardized by the coagulation management team. Monitoring was conducted weekly during the first 2 weeks of therapy and then every 2-4 weeks. The target INR range was 1.7‐2.5 for patients with valve replacement.

### Measures and Incentives

All participants received free pharmaceutical care and advice during the study period. Offline group participants were exempted from consultation fees in the anticoagulation pharmacy clinic during the study period. After the study, offline group participants were recommended to use the Alfalfa app. Registration and use of the Alfalfa app remained free for participants during and after the study. And the app also provided a free “anticoagulation circle” for patients to access educational resources of anticoagulation treatment and communicate with one another.

### Primary Outcome

The primary outcome was the percentage of TTR, calculated using the standard linear interpolation method [17]. TTR is a widely recognized measure of anticoagulant control quality, with TTR ≥60% indicating high-quality anticoagulation and TTR <60% indicating low-quality anticoagulation [18,19].

### Secondary Outcomes

#### Monitoring Frequency and INR Value Distribution

Monitoring frequency and INR distribution were assessed to evaluate compliance rates. According to the patient’s target range, INR values were categorized into 5 levels referring to previous studies [20,21]: extreme subtherapeutic, subtherapeutic, therapeutic, supratherapeutic, and extreme supratherapeutic. Definitions of these categories are provided in the Multimedia Appendix 1. Patients with multiple anticoagulation indications were assigned the highest INR target based on their conditions.

#### Clinical Events

The incidences of clinical events were used to evaluate safety differences between the 2 groups, including bleeding events, thrombotic events, and other related incidents. Bleeding events were classified as either minor or major bleeding events according to the International Society on Thrombosis and Haemostasis (ISTH) criteria. Minor bleeding events included epistaxis, gingival or oral bleeding, skin ecchymosis, fundus bleeding, increased or prolonged menstruation, hematuria and other bleeding symptoms that could be quickly stopped. Major bleeding events were defined as any bleeding requiring hospitalization or blood transfusion, including gastrointestinal bleeding and intracranial hemorrhage [22]. Thrombotic events included transient ischemic attack, ischemic strokes, venous thromboembolism and valve thrombosis. Other clinical events included warfarin-related emergency department visits and hospitalizations.

#### Costs

We conducted a cost analysis for both groups to demonstrate the economic differences observed during the follow-up period. The cost-effectiveness analysis was used to identify the most optimal solution. Economic indicators included the average cost per test, total cost per capita, average time per test, overall total cost, and the cost-effectiveness ratio. The average cost per test was defined as the mean expense per test per patient, including transportation costs. The total cost per capita referred to the average expense for all tests over a 3-month period per patient, also including transportation costs. The average time per test indicated the mean time required for 1 test per patient. The cost-effectiveness ratio was calculated as the ratio of total costs to efficiency, with costs including direct expenses such as INR testing fees and transportation expenses. A patient with TTR ≥60% was considered to have achieved an effective outcome. A lower cost-effectiveness ratio suggests that the intervention is more cost-efficient, indicating a more favorable and rational solution. All currency conversions in this paper are based on the exchange rate as of December 31, 2021.

#### Feasibility

Feasibility measures included acceptability and demand. Acceptability was measured through a satisfaction survey administered post intervention. A questionnaire designed by our previous study [23,24] was distributed to participants through WeChat in the form of a link to assess patient satisfaction and further demand for the Alfalfa app. Since the Alfalfa App did not support real-time one-on-one chats, we distributed survey links twice daily for 1 week in a WeChat group that included all participants. Completion of the surveys was voluntary, and each participant was allowed to submit only once (Multimedia Appendix 2). The surveys were distributed to all participants who had been using the Alfalfa App for at least 3 months (not limited to the web-based group). The percentage of responses for each question was calculated once the questionnaires were returned.

#### Data Collection

Basic information, including demographic characteristics of all participants, was collected from electronic medical records. The data automatically recorded in the Alfalfa app included duration of anticoagulation, target INR range, INR values and warfarin doses at each report, bleeding events, thromboembolic events, emergency department visits, hospitalizations, disease state, concurrent medications, and dietary habits. Information provided by clinical pharmacists, such as adjusted warfarin dosage, timing for subsequent INR tests, and recommendations on diet and exercise, was automatically collected in the Alfalfa app. Data from the offline warfarin management service, as well as costs for all participants, were collected from electronic medical records and telephone follow-ups.

### Statistical Analyses

Data were analyzed using Excel 2010 and SPSS (version 17.0, IBM Corp) statistical software. Different statistical methods were applied depending on the variable type results. An independent 2-sample, 2-tailed *t* test was used for statistical analysis of continuous variables, such as age, TTR, and costs. A chi-square test or Fisher exact test was used for categorical variables, such as sex, conditions, diseases, target INR, efficacy outcomes, adverse events, and INR results. Continuous variables are presented as mean (SD), while categorical variables are reported as frequency (percentage). Statistical significance was set at *P*<.05.

### Ethical Considerations

This study complied with the Declaration of Helsinki and was approved by the Ethics Committee of Fuwai Yunnan Cardiovascular Hospital, Kunming, Yunnan, China (Ethics No. 2020-010-01). It was also registered with the Chinese Clinical Trial Registry (ChiCTR1900021920). All participants provided written informed consent before the study and signed an electronic informed consent form again upon registering for the Alfalfa App. Participants were allowed to withdraw from the study at any time during the research process. All medical records and test reports of the participants were stored in the hospital where they received care, and personal privacy information was strictly protected. No additional compensation was provided to the patients for participating in this study; however, some free services were offered during the research period (see the *Measures and Incentives* subsection). The datasets generated or analyzed during the study are available from the corresponding author upon reasonable request.

## Results

### Participant Enrollment

**Figure 1.**
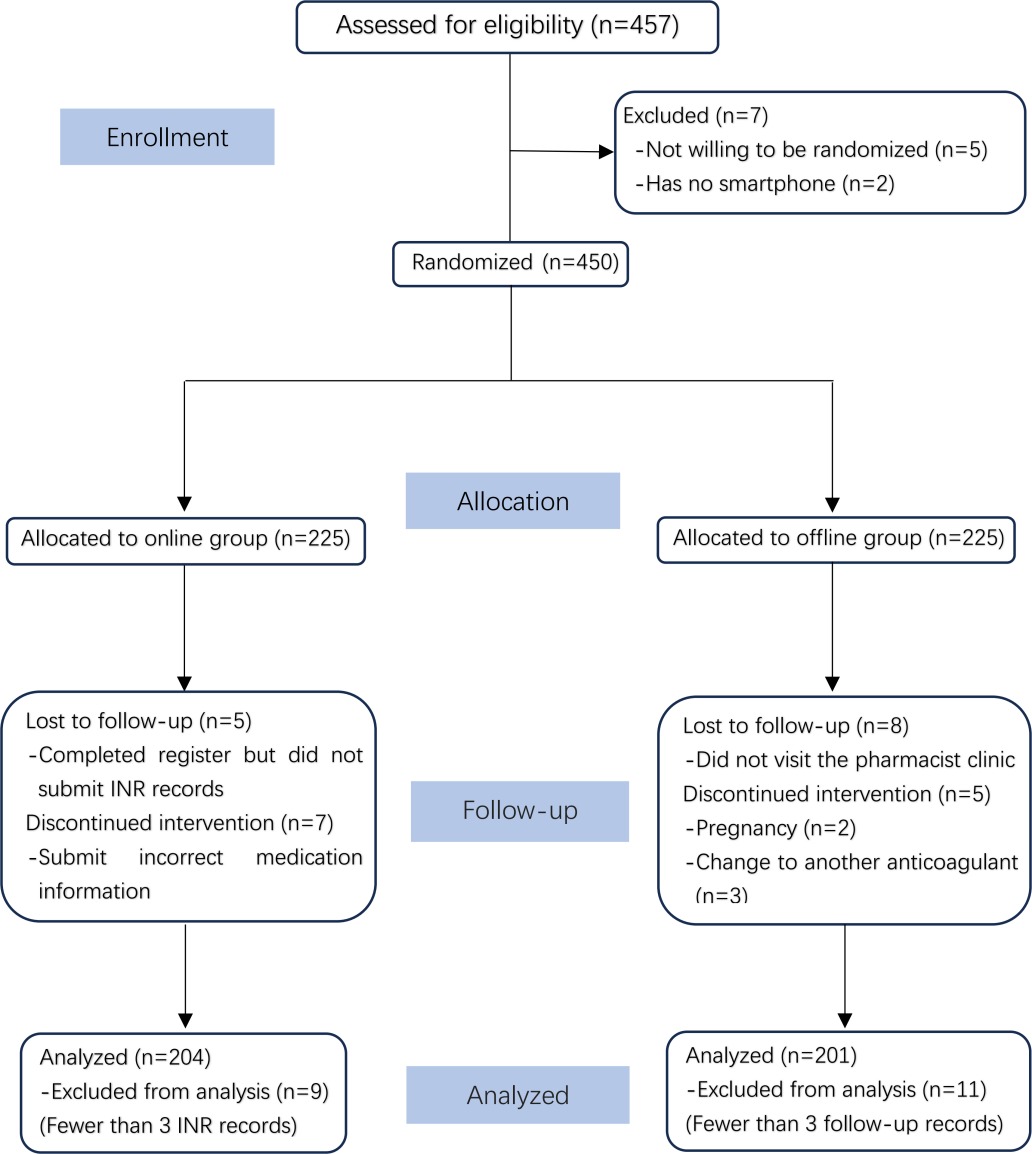
Enrollment flow chart. INR: international normalized ratio.

Between January 2020 and December 2021, a total of 457 patients from Fuwai Yunnan Cardiovascular Hospital were recruited for eligibility assessment, and 450 were enrolled in the study. Of these, 225 participants were randomized to the web-based group and 225 to the offline group. Participants were excluded if they completed registration but did not submit INR records (n=5), did not visit the pharmacist clinic (n=8), provided incorrect medication information (n=7), were pregnant (n=2), switched to another anticoagulant (n=3), or had fewer than 3 follow-up visits or INR records (n=20). This resulted in a total of 405 participants included in the final analysis (Figure 1). There were no missing data among these participants, and baseline characteristics were comparable across study groups (Table 1).

**Table 1.** Patients’ characteristics.

Characteristics	Web-based (n=204)	Offline (n=201)	*P* value
Age (years), mean (SD)	49.66 (12.37)	51.97 (13.58)	.07
Sex (male), n (%)	115 (56.4)	101 (50.2)	.22
Height (cm), mean (SD)	163.2 (7.91)	161.78 (9.78)	.11
Weight (kg), mean (SD)	62.95 (15.53)	61.12 (13.38)	.21
Baseline INR, mean^a^ (SD)	1.09 (0.13)	1.11 (0.14)	.14
Diseases, n (%)			
Hypertension	33 (16.18)	31 (15.42)	.29
Diabetes	8 (3.92)	7 (3.48)	.12
Hyperuricemia or gout	2 (1.0)	1 (0.5)	.99
Co-antiplatelet therapy	14 (6.86)	21 (10.45)	.20
Smoking, n (%)	66 (32.4)	59 (29.4)	.51
Alcohol intake, n (%)	20 (9.8)	26 (12.9)	.32
Regional Distribution, n (%)			.50
Kunming	70 (34.3)	64 (31.8)	
Prefecture-level cities	128 (62.8)	134 (66.7)	
Outside Yunnan province	6 (2.9)	3 (1.5)	

a INR: international normalized ratio.

### Primary Outcomes (TTR)

Table 2 shows that the mean TTR was significantly higher in the web-based group compared with the offline group (66.46% vs 46.65%, *P*<.001). In addition, the percentage of INR values within the therapeutic range was higher in the web-based group compared with the offline group (124/204, 60.78% vs 67/201, 33.33%; *P*<.001; Table 2).

**Table 2.** Percentage of time in therapeutic range per study group.

Variable	Web-based (n=204)	Offline (n=201)	*P* value
TTR^a^ (%), mean (SD)	66.46 (25.22)	46.65 (39.13)	<.001
Control ratio, n (%)			<.001
TTR≥60%	124 (60.78)	67 (33.33)	
TTR<60%	80 (39.22)	134 (66.67)	

a TTR: time in therapeutic range.

### Secondary Outcomes

#### Monitoring Frequency and INR Value Distribution

The Alfalfa app group had a higher monitoring frequency compared to the offline group (8.14 vs 4.47, *P*<.001) and a greater percentage of INR values within the therapeutic range (896/1660, 53.98% vs 346/899, 38.49%; *P*<.001). Participants managed through the Alfalfa app also had lower rates of subtherapeutic INR values (235/1660, 14.16% vs 152/899, 16.91%; *P*<.05), and extreme subtherapeutic INR values (273/1660, 16.45% vs 186/899, 20.69%; *P*<.05) compared with those managed by offline services. The incidence of supratherapeutic and extreme supratherapeutic INR values was similar between the 2 groups (Table 3).

**Table 3.** Monitoring frequency and distribution of international normalized ratio values.

Variable	Web-based (n=204/1660)	Offline (n=201/899)	*P* value
Monitoring frequency (times), mean (SD)	8.14 (4.75)	4.47 (3.84)	<.001
Extreme subtherapeutic, n (%)	273 (16.45)	186 (20.69)	.003
Subtherapeutic, n (%)	235 (14.16)	152 (16.91)	.02
Therapeutic, n (%)	896 (53.98）	346 (38.49)	<.001
Supratherapeutic, n (%)	251 (15.12)	204 (22.69)	.11
Extreme supratherapeutic, n (%)	5 (0.3)	10 (1.11)	.25

#### Clinical Events

The incidence of minor bleeding events vs was higher in the Alfalfa app group compared with the offline management group (12/204, 5.9% vs 3/201, 1.5%; *P*=.02). However, there were no significant differences between the groups in major bleeding events (3/204, 1.5% vs 1/201, 0.5% *P*=.63), thromboembolic events (1/204, 0.5% vs 0/201, 0%; *P*=.99), warfarin-related emergency department visits (1/204, 0.5% vs 2/201, 1.0%; *P*=.99) or warfarin-related hospital admissions (3/204, 1.5% vs 2/201, 1.0%; *P*=.99; Table 4).

**Table 4.** The incidences of clinical events.

Variable	Web-based (n=204)	Offline (n=201)	*P* value
Minor bleeding, n (%)	12 (5.9)	3 (1.5)	.02
Major bleeding, n (%)	3 (1.5)	1 (0.5)	.63
Thromboembolic, n (%)	1 (0.5)	0 (0)	.99
Warfarin-related emergency department visits, n (%)	1 (0.5)	2 (1)	.99
Warfarin-related hospital admissions, n (%)	3 (1.5)	2 (1)	.99

#### Costs

The *t* test revealed that the average cost per test (42.37 vs 78.3, *P*<.001) and the average time per test (47.42 vs 90.74, *P*<.001) were significantly lower for the web-based group compared with the offline group. While the total cost per capita was similar between the 2 groups (234.55 vs 220.98, *P*=.58), the cost-effectiveness ratio was lower for the web-based group (385.9 vs 662.9; Table 5).

**Table 5.** Economic differences.

Variable	Web-based (n=204)	Offline (n=201)	*P* value
Average cost per test, mean (SD)	42.37 (31.6)	78.3 (77.91)	<.001
Total cost per capita, mean (SD)	234.55 (270.25)	220.98 (224.74)	.58
Average time per test, mean (SD)	47.42 (41.01)	90.74 (74.57)	<.001
Total cost	¥47848.7 (US $7502.68)	¥44417.6 (US $6964.68)	—^a^
Effective, n (%)	124 (60.8)	67 (33.3)	—
Cost-effect ratio (C/E; as in Table 1)^b^	385.9	662.9	—

a Not applicable.

b C/E: total cost/effective value.

### Acceptability and Demand

After 1 week of distributing and reminding participants to complete the survey links, a total of 86 voluntarily completed questionnaires were collected. Of these, 59.3% (51/86) had used the Alfalfa app for over a year, and 77.9% (67/86) preferred to stay in contact with their doctor through the app. The vast majority (83/86, 96.5%) found the app convenient, noting the ability to receive timely responses from doctors without needing to visit the hospital and to view all their report records within the app. Approximately 80% (69/86) of the patients expressed positive experiences with the doctor’s responses (82/86, 95.3%), the completeness of records (75/86, 87.2%), and the free services (69/86, 80.2%).

All participants were satisfied with warfarin dose adjustment (100%, 86/86), timely responses from doctors (86/86, 100%), the report submission process (78/86, 90.7%), and the history records functions in the app (85/86, 98.8%). Nearly half of the respondents (42/86, 48.8%) indicated they would be willing to pay a fee ranging from ¥3 (US $0.4704) to ¥10 (US $1.568) for the app’s services. A large majority (77/86, 89.5%) expressed a preference for services provided by experts with senior professional titles. A few participants (10/86, 11.6%) suggested improvements, such as simplifying the app’s operation, adding a feature for video interactions with doctors (3/86, 3.49%), and establishing a more comprehensive medical history record function (1/86, 1.16%; Multimedia Appendix 2).

## Discussion

### Principal Findings

The main goal of this study was to evaluate the efficacy, safety, and economic impact of using the Alfalfa app, a smartphone app designed for dose consultation and adjustment in patients undergoing long-term anticoagulation therapy with warfarin. TTR was selected as the primary outcome, with a TTR of 60% or higher generally considered indicative of high-quality anticoagulant therapy [25]. In our study, TTR was significantly higher in the Alfalfa app group compared with the offline group (66.46% vs 46.65%, *P*<.001). While a study by Lee et al [26] reported contrary findings, our results align with the majority of telemedicine-based anticoagulation studies, which show improved TTR outcomes compared with traditional management methods [27-32]. Previous cohort studies in Eastern China also supported the efficacy of the Alfalfa App, reporting significantly higher TTRs in their intervention groups (61% and 79.35%, respectively) [15,33]. Our findings further confirmed the effectiveness of Alfalfa App in improving the quality of anticoagulation management.

Our research also demonstrated that participants in the web-based group had a significantly higher frequency of INR monitoring compared with those in the offline group (8.14 vs 4.47, *P*<.001). This indicates that the Alfalfa App facilitated better interaction between patients and health care providers, enabling participants to easily upload their INR records and receive timely feedback from clinical pharmacists. In addition, the app’s supervision and tracking features likely contributed to the regular monitoring observed. These findings are consistent with 2 other retrospective cohort studies, which also reported higher rates of therapeutic INR values in the web-based group [15,33]. However, compared with the other 2 retrospective studies, there was no consistent pattern in the distribution of extreme subtherapeutic, subtherapeutic, supratherapeutic, and extreme supratherapeutic INR values between the groups. The result indicates that the web-based management model helps improve the percentage of INR values within the therapeutic range (indirectly equivalent to TTR), but when the INR values fall outside the therapeutic range, they do not necessarily tend to be too high or too low. This suggests that there may be no direct association between INR value distribution and anticoagulation management methods, indicating a need for further exploration with larger sample sizes.

Our research also revealed the higher incidence of minor bleeding events in the Alfalfa app group, consistent with the results reported in a similar cohort study [15]. According to a meta-analysis of 12 randomized controlled trials, telemedicine combined with portable coagulometers significantly improved TTR and reduced the incidence of thrombotic events in cardiovascular patients [34]. However, several studies reported no statistically significant difference (*P*>.05) in bleeding or thrombotic events between the eHealth tools group and the conventional management groups [28,30,35]. eHealth tools can be broadly categorized into 4 types, namely computer-based support systems, electronic health records, telemedicine platforms, and mobile apps. Among these, mobile apps offer the greatest convenience, allowing patients to communicate with doctors or pharmacists at any time [36]. This may explain the higher detection rate of minor bleeding events in the Alfalfa app group, as patients were more likely to report symptoms to clinical pharmacists through the app in time, especially during the period of COVID-19 pandemic, when patients were less willing to go out for medical treatment. This made minor bleeding in the web-based group of the study easier to detect. The pandemic also limited our ability to fully assess clinical event outcomes due to the reduced sample size and limited participant enrollment.

Our findings also suggest that the Alfalfa App may reduce the cost per visit for anticoagulation management and save participants time in return visits. The convenience of using the app led to higher adherence to follow-up visits in the web-based group, resulting in a greater number of return visits per capita during the follow-up period. Although we did not calculate the incremental cost-effectiveness ratio due to a lack of authoritative data, the cost-effectiveness value suggests a positive economic outlook for the Alfalfa App. It should be noted that patients in both the web-based and offline groups were exempt from registration fees. If web-based services were to be charged, combined with the increased number of follow-up visits, this could potentially offset the cost savings from not visiting the hospital, and the overall expenses might even be higher [37], and more research is needed to explore the full economic impact of the app.

Two studies revealed that most patients were satisfied with the use of eHealth tools, which also improved their quality of life [38,39]. Consistent with these findings, our survey results of the experience and satisfaction with pharmaceutical care and Alfalfa App also showed that the vast majority of patients were highly satisfied with the dose adjustment, timely responses, and free service provided. The timeliness and convenience of the Alfalfa App were particularly appreciated. However, a small number of participants complained the app difficult to use. During the follow-up, 5 participants discontinued to submit their results, resulting in withdrawals. This suggests that both the app and anticoagulant management services have areas for improvement. With the advancements of machine learning, the integration of deep learning, reinforcement learning, and ensemble learning in warfarin management after heart valve surgery is becoming more prevalent [40]. Internet+pharmacy care through app has the potential to enhance efficiency, reduce time and economic costs, and improve the quality of medical care, especially by reducing the disparities among primary care providers.

### Limitations

Although our study demonstrates promising results regarding the efficacy of internet+pharmacy care through Alfalfa Appin improving anticoagulant quality among patients following heart valve surgery, several limitations must be acknowledged. First, because the study’s enrollment period overlapped with the COVID-19 lockdown, patients reduced their hospital visits. The significant decline in hospital patient flow from 2020 to 2022 confirms this trend. As a result, the study’s sample size was limited, preventing us from conducting subgroup analyses. Second, this was a single-center study conducted in southwestern China, which may limit the generalizability of the findings to other populations. Further prospective, randomized, multicenter studies are needed to confirm these results.

We estimated a sample size of 446, assuming a low dropout rate of 10%. By the end of the study, 405 participants were included in the analysis, and the actual dropout rate was within expectations. With strong effect sizes, we maintained adequate statistical power. However, due to limitations with the Alfalfa App, we were unable to send private information to participants, which prevented us from distributing the satisfaction questionnaire individually. As a result, only 86 questionnaires were collected. In the future, we aim to address this limitation to enhance the completeness of research data.

### Conclusions

In conclusion, the “Internet+Pharmacy Care” model through the Alfalfa App can improve the quality of warfarin anticoagulation in patients after heart valve surgery. Compared with traditional outpatient service, web-based management does not increase the risk of major bleeding, embolism, or hospitalization events, and is both economical and cost-effective. Looking forward, we anticipate that machine learning and artificial intelligence will bring new opportunities for innovation in anticoagulation management. Future studies with larger sample sizes, multicenter designs, and long-term follow-up are needed to further validate the role of these technologies.

## Supplementary material

10.2196/53586 Multimedia Appendix 1 The classification criteria for international normalized ratio.

10.2196/53586 Multimedia Appendix 2 Alfalfa app user satisfaction survey and survey results of Alfalfa app usage.

10.2196/53586 Checklist 1 CONSORT (Consolidated Standards of Reporting Trials) checklist.

## Abbreviations

INR: international normalized ratio
ISTH: International Society on Thrombosis and Haemostasis
TTR: time in therapy range
