# Supplementary material for: Efficacy, Safety, and Cost-Effectiveness of “Internet + Pharmacy Care” Via the Alfalfa App in Warfarin Therapy Management After Cardiac Valve Replacement: Randomized Controlled Trial
Source: JMIR Mhealth Uhealth. 2025 May 20;13:e53586. doi: 10.2196/53586 (PMC12112255; doi:10.2196/53586)
Supplement: Multimedia Appendix 1 [file mhealth-v13-e53586-s001.docx]

**Multimedia Appendix 1**

The Classification Criteria for International Normalized Ratio

| INR classification | Definition |
| --- | --- |
| Severe subtherapeutic range | INR＜( Lower limit of the target range-0.2 ) |
| Subtherapeutic range | ( Lower limit of the target range-0.2)≤ INR＜ Lower limit of the target range |
| Therapeutic range | Lower limit of the target range≤ INR ≤ Upper limit of target range |
| Supratherapeutic range | ( Upper limit of target range+0.2 )＜ INR ≤4.5 |
| Severe supratherapeutic range | INR ＞4.5 |
