# Supplementary material for: Efficacy, Safety, and Cost-Effectiveness of “Internet + Pharmacy Care” Via the Alfalfa App in Warfarin Therapy Management After Cardiac Valve Replacement: Randomized Controlled Trial
Source: JMIR Mhealth Uhealth. 2025 May 20;13:e53586. doi: 10.2196/53586 (PMC12112255; doi:10.2196/53586)
Supplement: Multimedia Appendix 2 [file mhealth-v13-e53586-s002.docx]

**Multimedia Appendix 2-1**

**Alfalfa App User Satisfaction Survey**

To further improve our coumarin anticoagulant guidance service, we kindly request a few minutes of your time to provide feedback on your experience with the Alfalfa App. Your valuable insights are greatly appreciated, and we look forward to your participation!

**General Information:**

1. Your name?
2. Your gender?

A. Male

B. Female

3. Your age?

A. 20-45 years

B. 45-65 years

C. 65-75 years

D. Over 75 years

4.How long have you been using the Alfalfa App?

A. 1-3 months

B. 3-6 months

C. 6-12 months

D. Over 1 year

E. Never used

5. During prolonged use of coumarin, which method do you prefer to stay in contact with your doctor? (Multiple choices allowed)

A. Face-to-face consultation

B. Phone/doctor's private WeChat

C. Consultation in a WeChat group

D. Alfalfa App

6. Why do you prefer face-to-face consultations?

A. Comprehensive assessment by the doctor and easier communication

B. Inconvenient, requires hospital visits, rarely choose face-to-face consultation

7. Why do you prefer consultation through phone/doctor's private WeChat?

A. Trust the doctor who is familiar with my case, more convenient without hospital visits

B. Doctor is usually busy, rarely use doctor's private WeChat

8. Why do you prefer consultation in a WeChat group?

A. Convenient, no hospital visit, and timely responses from dedicated doctors in the group

B. Difficult to browse past records in the group, rarely use group consultation

9. Why do you prefer the Alfalfa App?

A. Convenient, no need for hospital visits, dedicated doctors respond promptly in the app, can view all reported records, easier continuous monitoring for both patient and doctor

B. Difficult to use, don't use the app

**App Satisfaction Levels:**

10. What is your best experience using the Alfalfa App? (Multiple choices allowed)

A. Prompt response from doctors

B. Comprehensive record of medication dosage and test results

C. Offers free services

11. How do you feel about the warfarin dose adjustments in the Alfalfa App?

A. Dose adjustments are accurate, very satisfied

B. Dose adjustments are mostly accurate, satisfied

C. Dose adjustments are often inaccurate, neutral

D. Dose adjustments are not accurate, unsatisfied

12. How timely do you find the clinical pharmacists' responses in the Alfalfa App?

A. Extremely prompt, very satisfied

B. Generally prompt, satisfied

C. Slow, neutrals

D. Very slow, unsatisfied

13. How do you feel about viewing medication and test records in the Alfalfa App?

A. Able to review past records at any time, very satisfied

B. Most of the time the records can be queried, satisfied

C. Don't usually check past records, neutral

D. Could not get the record, unsatisfied

14. How was your experience with the reporting and submission process in the app?

A. you can learn to use it yourself without instruction, very satisfied

B. Simple to use with instruction, satisfied

C. Somewhat difficult, neutral

D. Very complicated, unsatisfied

15. How do you feel about the app being free of charge?

A. Free and very satisfied

B. Free and satisfied with the service, satisfied

C. Free, but average service, neutral

D. Free, but poor service, unsatisfied

**Suggestions for App Improvement:**

16. If a fee were charged, what price range would you accept?

A. 3-10 yuan/instance

B. 10-50 yuan/instance

C. 100-200 yuan/year

D. Would not use if charged

17. What professional title do you prefer for the consulting doctor?

A. Junior B. Intermediate C. Senior (Chief/Deputy Chief)

18. What are the aspects you find lacking or inconvenient while using the Alfalfa App? (Multiple choices allowed)

A. Difficult reporting procedure

B. Inability to have face-to-face consultations

C. Medication queries are not specifically addressed

19. Do you have any further suggestions for the Alfalfa App?

**Multimedia Appendix 2-2**

**Survey Results of Alfalfa App usage**

| **Items** | **Number of Users** | **Percentage** |
| --- | --- | --- |
| **Survey Results of Alfalfa App usage** |  |  |
| **Duration of app Usage** |  |  |
| 1-3 months | 9 | 10.50% |
| 3-6 months | 9 | 10.50% |
| 6-12 months | 13 | 15.10% |
| 1 year and above | 51 | 59.30% |
| Never used | 4 | 4.70% |
| **Preferred Method of Contact with Doctors** |  |  |
| Face-to-face outpatient consultation | 23 | 26.70% |
| Phone/Private WeChat of the doctor | 32 | 37.20% |
| Consultation in WeChat group | 56 | 65.10% |
| Alfalfa Health Management Public Account | 67 | 77.90% |
| **Reasons for Using the Alfalfa app** |  |  |
| Convenient, no need to visit the hospital | 83 | 96.50% |
| Difficult to use, don't use the app | 3 | 3.50% |
| **Best Features of the Alfalfa app** |  |  |
| Timely responses from doctors | 82 | 95.30% |
| Comprehensive records | 75 | 87.20% |
| Offers free services | 69 | 80.20% |
| **Satisfaction Survey** |  |  |
| **Experience Adjusting Warfarin Dosage** |  |  |
| Very Satisfied | 68 | 79.1% |
| Satisfied | 18 | 20.9% |
| Neutral | 0 | 0% |
| Unsatisfied | 0 | 0% |
| **Experience with Timely clinical pharmacist Responses** |  |  |
| Very Satisfied | 82 | 95.3% |
| Satisfied | 4 | 4.7% |
| Neutral | 0 | 0% |
| Unsatisfied | 0 | 0% |
| **Experience with Historical Records Function** |  |  |
| Very Satisfied | 85 | 98.8% |
| Satisfied | 0 | 0% |
| Neutral | 1 | 1.2% |
| Unsatisfied | 0 | 0% |
| **Experience with Reporting Procedure** |  |  |
| Very Satisfied | 0 | 0% |
| Satisfied | 78 | 90.7% |
| Neutral | 7 | 8.1% |
| Unsatisfied | 1 | 1.2% |
| **Experience with Free Service** |  |  |
| Very Satisfied | 0 | 0% |
| Satisfied | 83 | 96.5% |
| Neutral | 3 | 3.5% |
| Unsatisfied | 0 | 0% |
